# Using bodily displays to facilitating approach action outcomes within the context of a personally relevant task

**DOI:** 10.1002/brb3.2855

**Published:** 2022-12-26

**Authors:** Emma Elkjær, Mai B. Mikkelsen, Gitte Tramm, Johannes Michalak, Douglas S. Mennin, Mia S. O'Toole

**Affiliations:** ^1^ Department for Psychology and Behavioral Sciences Aarhus University Aarhus Denmark; ^2^ Department of Psychology and Psychotherapy, Witten/Herdecke University Witten Germany; ^3^ Department of Psychology, Teachers College Columbia University New York New York USA

**Keywords:** bodily displays, contractive displays, embodiment, emotion regulation, expansive displays

## Abstract

**Background:**

Considerable attention has been paid to the effect of bodily (expansive and contractive) displays on affective, behavioral, and hormonal outcomes. However, the majority of past studies are limited by a lack of control groups with neutral displays and low personal relevance of the experimental tasks employed. The present study aimed to investigate the effect of adopting different bodily displays, including neutral displays, within the context of a personally relevant task.

**Methods:**

In an experiment with healthy participants (*N* = 90), we investigated the effects of two different bodily manipulations (i.e., expansive and contractive), compared to a control group (i.e., neutral displays). Effects were evaluated in terms of completed valued action in addition to processes considered potentially helpful in preparing and motivating the individual to take valued action, including a change in emotion experience, action tendencies, and appraisals.

**Results:**

Several main effects were detected and only few significant interaction effects were revealed. In case of group differences, results showed that expansive bodily displays outperformed the control group, leading to more positive emotions, more approach action tendencies, less negative emotion variability, and less avoidance action tendencies toward threat.

**Discussion:**

These results mainly suggest that identifying a valued action and explicating the underlying motivational conflict may be beneficial regardless of bodily displays. This conclusion runs somewhat counter both to our hypotheses and to findings in recent meta‐analytic work. However, previous experiments have not evaluated the effect of bodily displays within a personally relevant context.

## Graphical abstract

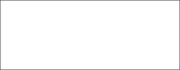

## 1 INTRODUCTION

From an embodiment perspective, affective processes and perception of action possibilities are guided, shaped, and constrained by our bodily engagement with the world (Fuchs et al., 2012; Niedenthal et al., 2005; Winkielman et al., 2014). As such, changes in bodily displays (i.e., posture and movement) may alter this engagement and are therefore believed to hold a regulatory potential for facilitating action (Cesario & Mcdonald, 2013; Elkjær et al., 2022; Veenstra et al., 2017; Yap et al., 2013). A massive interest in bodily displays has sprung up as a result of a cardinal study from 2010, reporting that it was possible to alter not only affective states and behavior but also hormone levels by assuming an expansive display (so‐called power posing; Carney et al., 2010).

Studies of bodily displays have frequently compared two sets of displays: expansive displays, which have often been associated with approach and engagement, and contractive displays, commonly associated with avoidance or withdrawal (O'Toole & Mikkelsen, 2021). Recently, a number of reviews and meta‐analyses have been conducted on the effect of expansive and contractive displays on affective, behavioral, and hormonal outcomes (Carney et al., 2015; Cesario et al., 2017; Cuddy et al., 2018; Elkjær et al., 2022; Körner & Schütz, 2020; Körner et al., 2022; Simmons & Simonsohn, 2017). These syntheses find that engaging in either expansive or contractive bodily displays alters affective (e.g., power feelings, self‐efficacy) and behavioral outcomes (*g* = 0.36, *p* = < .001; Elkjær et al., 2022) but not hormonal outcomes (i.e., testosterone, cortisol). Three critical limitations in the current body of literature have been identified. These include (1) a lack of control groups—both passive control groups (e.g., passive or usual bodily displays) and active control comparisons (e.g., nonspecific bodily activity), (2) limited knowledge of the effect of bodily displays during personally relevant situations, and (3) limited knowledge of the effect of bodily displays on specific outcomes believed to be involved in action facilitation.

First, regarding the shortage of experimental studies including a neutral control condition, this methodological issue limits the ability to ascribe a possible effect of expansive or contractive bodily displays to the appropriate condition. Indeed, recent reviews and meta‐analyses found that only a small number of studies compared expansive or contractive bodily displays to a neutral condition (Elkjær et al., 2022; Körner et al., 2022). When investigating the effects of these studies on affective, behavioral, and hormonal outcomes, contractive displays differed from neutral displays, while expansive displays did not differ from neutral displays. While this appears to indicate that the absence of contractive displays is more central to these outcomes than the presence of expansive displays, the small number of studies makes definitive conclusions difficult.

Second, to date, most studies have relied on experimentally manipulated tasks or goals (e.g., simulated job interviews or gambling tasks), without accounting for the individual's own goals and the resulting perceived relevance of the experimental task. One could imagine a bodily display being congruent with an experimental task (e.g., expansive displays believed to foster power feelings in a mock job interview) but incongruent with the individual's current goal (e.g., being happy with current “real” job, but wanting to flee the experimental situation concerning the “mock” job due to intense fear). Such instances could potentially mask the adaptive value of the bodily display. Thus, when our recent meta‐analysis suggests, albeit on a small number of studies, that the absence of contractive displays and movement is more important than the presence of expansive displays and movement (Elkjær et al., 2022), such finding may be a context‐dependent finding, attributable to the limited personal relevance of the experimentally manipulated task. In order to obtain a more ecologically valid result of adopting different displays, studies should take into account the personal relevance.

Third, while previous studies have evaluated the effect of bodily manipulations on both both behavior and specific emotions or mood (e.g., Cuddy et al., 2018), less attention has been paid to evaluating *how* bodily manipulations may be involved in action facilitation. Derived from current embodiment and emotion theories (Frijda et al., 1989; Houben et al., 2015; Niedenthal, 2007; Niedenthal et al., 2005; Scherer & Moors, 2019; Winkielman et al., 2014), three outcomes may be particularly important to explore in this regard: *emotion experience* (e.g., emotion variability), *appraisals* (i.e., perceived ability to handle the situation), and *action tendencies* (i.e., approach and avoidance impetuses).

First concerning emotion experience, research into the effect of expansive and contractive bodily displays has mainly focused on changes in either single emotions or overall mood. Lacking from this research is a focus on the effect of these displays on *how* the individual experiences their emotions. Indeed, the ability to respond adaptively to an emotion‐inducing situation has been proposed to largely depend on the individual's emotion experience (Grossmann et al., 2016; Lindquist & Barrett, 2010; O'Toole et al., 2020). One frequently investigated indicator of emotion experience is emotion variability (EV; the dispersion of emotions and variation in their intensities, typically operationalized as the standard deviation around the person's mean of either negative or positive emotions; Houben et al., 2015; Kalokerinos et al., 2020; Kuppens et al., 2007). High levels of EV in emotion experiences have consistently been associated with maladaptive psychological functioning, hypothesized to be due to EV capturing emotional overload or instability. However, these findings concern the person level (i.e., trait or average measures) and research is sparse at the situational level (i.e., state or momentary measures; Houben et al., 2015; Kuppens et al., 2007; O'Toole et al., 2020). Rooted in the assumption that emotions are functional, the ability to hone or prioritize the most helpful emotions in a given situation—experienced as *less* EV—could be an important step in taking adaptive action (O'Toole et al., 2020). Albeit speculative given a lack of previous empirical studies, bodily displays may be hypothesized to assist the individual in narrowing the emotional scope. Indeed, holding a particular bodily display (e.g., expansive) in the face of a reward may be hypothesized to guide the individual's attention toward emotions associated with approaching the reward more so than other postures (e.g., contractive). However, it remains to be investigated (1) if EV changes in response to bodily manipulations, and (2) if such changes differ between expansive and contractive displays.

Second, another factor that may be hypothesized to be involved in the individual's perception of action possibilities and ultimately choice of action is the individual's *appraisals* of a given situation (e.g., cognitive evaluations of motivational relevance, difficulty of action, action self‐efficacy). In contemporary appraisal‐based accounts of emotions (Frijda, 2007; Scherer & Moors, 2019), emotions are considered “meaning detectors,” involving appraisals of the personal relevance of a situation based on the individual's current goals. If changes in bodily displays alter the way in which an individual engages with the world, such displays may change the individual's interpretation of the situation, including goals and action possibilities (Gross, 2014; Scherer & Moors, 2019). Appraisals related to the perceived motivational relevance, difficulty of accomplishing this goal, and the action self‐efficacy appear particularly relevant for facilitating action.

Lastly, another outcome relevant for the assessment of action is the individual's *action tendencies*. Action tendencies have been defined as “readiness to engage in action and for establishing, maintaining or breaking the relation with particular aspects of the environment” (Frijda, 1987, p. 132), which may or may not turn into actual over behavior. Two recent studies indicate that it is indeed possible to alter action tendencies. One study showed that it was possible to increase approach motivation by leaning forward in a so‐called “high posture condition” (Miragall et al., 2021). The other study showed that it was only possible to change avoidance tendencies with contractive (and not expansive) displays (Metzler et al., 2020). More knowledge is needed on when and how action tendencies can be altered by means of bodily displays. In the present study, we work from the hypothesis that expansive displays will be associated with more approach action tendencies, whereas contractive displays will be associated with more avoidance/withdrawal impetuses.

### 1.1 The present study

We wanted to investigate how the manipulation of bodily displays woould impact the individual's response to a personally relevant motivational conflict. In doing this, we compared expansive, contractive, and neutral displays. Effects were evaluated in terms of completed valued action in addition to specific processes considered potentially helpful in preparing and motivating the individual to take the valued action, that is, emotion experience (i.e., EV), appraisals (i.e., cognitive evaluations of motivational relevance, difficulty of action, and action self‐efficacy), and action tendencies. We expected the group assuming expansive displays [EXP] to (1) show higher self‐efficacy in taking valued action, (2) appraise the action as more important, (3) experience lower difficulty of initiating action, (4) show more approach action tendencies, (5) experience less negative EV, and (6) be more likely to take valued action compared both with the group assuming contractive displays [CON] and the neutral (N) control condition. We also explored if the CON and N conditions would differ from each other.

The motivational conflict was operationalized as an approach/avoidance or approach/withdrawal conflict. Approach involved a future‐oriented action that the individual found valuable to take (e.g., reaching out to a friend, going to the gym, pick child up early), where avoidance and/or withdrawal involved the feeling of wanting to flee or escape from the situation, being held back by anxiety or worry, or difficulty initiating the action because of low mood or energy.

## 2 METHODS

The project was registered and approved by the Danish Data Protection Agency at Aarhus University and was preregistered at *As Predicted* (#41407). See https://aspredicted.org/blind.php?x=9j3b38.

### 2.1 Participants

One‐hundred participants were recruited through social media and various departments at Aarhus University. Participants were required to be adult (≥18 years old) and proficient in Danish. The study was conducted according to the 1964 Declaration of Helsinki, and informed oral and written consent was obtained from all research participants. Based on a priori power calculations, using a repeated‐measures ANOVA interaction analysis, 2 (time; before vs. after the manipulation) × 3 (condition; EXP, CON, N), 90 participants were required to detect a small effect size (*d* = 0.34), with an alpha of .05 and a beta of .20 (G*power 3.0.10; Faul et al., 2007).

Participants were excluded if they did not want to complete the experiment due to self‐reported distress (e.g., elevated anxiety in response to motivational conflict) or could not identify an appropriate motivational conflict (i.e., a perceived difficulty level for taking the identified action of ≥5). Ten participants were excluded on this basis, leaving the final sample at the target of 90 (77.8% women, mean age = 24.43, *SD* = 3.36). Most participants were psychology students (37.8%), 15.6% were medical students, 15.6% were unemployed, 12.2% were university students from various other departments than psychology and medicine, 8.9% were employed full‐time, 4.4% were in high schools, 2.2% were in youth educations, 2.2% were in medium‐cycle higher education programs, and 1.1% were PhD students. Participants were compensated with a gift voucher of 90 DKK for their participation (∼15 USD).

### 2.2 Procedures

The experiment took place online as a video meeting (via the software program “Zoom”) lasting approximately 1 h. The online format was chosen due to pandemic‐related restrictions, where the video format allowed the experimenter to ensure instruction following. All questionnaires were completed online using the software package SurveyXact. Upon having obtained oral and written consent, all participants completed a baseline survey. Participants were then invited to nominate a valued action that they could potentially take within the next week or two. The action was set up within the context of a motivational conflict, where the participant (1) wanted to or hoped they could take the action (i.e., approach) because it was perceived as important or meaningful according to their values but (2) at the same time feeling held back by anxiety/worries and/or low mood or lack of energy (i.e., avoidance). Examples of motivational conflicts included wanting to ask out a potential partner or to apply for a new job, but being held back by anxiety and fear of rejecting as well as wanting to go to the gym, but lacking the motivation or energy to do so. Subsequently, participants completed a questionnaire on current emotions, appraisals, and action tendencies in relation to this action. Participants were then assigned to one of three manipulations via a computer‐generated randomization list: (1) an expansive body display manipulation (i.e., EXP, *N* = 30), (2) a contractive body display manipulation (i.e., CON, *N* = 29), or (3) a neutral control condition (*N* = 31). The three manipulations were inspired by previous research on bodily displays (Carney et al., 2010; Cuddy et al., 2015) and matched on duration (i.e., 4 min). The bodily exercises (i.e., EXP + CON) included the same duration of standing up and sitting down. Online supervision during the manipulation ensured that participants adopted the appropriate display. In order to accommodate demand characteristics, participants in the bodily conditions were told that the study was about physiology and action, testing the effect of physical activities known to affect the pulse in certain ways. At the end of the online session, a follow‐up phone call was scheduled within the following 1–2 weeks. During the phone call, participants were debriefed and asked if they completed the identified valued action. In addition, we asked participants if they were familiar with research on embodiment and well‐being.

### 2.3 Manipulations

The *EXP* regulation strategy was based on research demonstrating how these displays are associated with more positive mood and more overt approach behavior (Carney et al., 2010; Elkjær et al., 2022; Körner et al., 2022). The *CON* regulation strategy was similarly based on research demonstrating that these displays are associated with less empowerment and less overt approach behavior (Carney et al., 2010; Yap et al., 2013). Both manipulations involved instructions to change posture (e.g., sitting up straight) and movement (e.g., nodding) in an attempt to maximize the potential effect. The neutral control condition was included to make sure that effects were assigned to the appropriate condition and to control for experimental demands (Cesario & Johnson, 2018). All participants were asked to listen to a bodily instruction, which was read aloud. See Appendix S1 for the full scripts.

### 2.4 Materials

#### 2.4.1 Baseline measures

*Psychological distress* was assessed with the Hospital Anxiety and Depression Scale (HADS; Zigmond & Snaith, 1983) and *satisfaction with life* was measured with the Satisfaction With Life Scale (SWLS; Diener et al., 1985). *Positive* and *negative emotions* were assessed with the trait version of the Positive and Negative Affective Scale (PANAS; Watson et al., 1988).^i^

#### 2.4.2 Outcome measures

*Emotions* were assessed with the state PANAS, inquiring about momentary levels of 10 positive and 10 negative emotions (Watson et al., 1988). All emotions were rated on a 5‐point Likert scale. EV was calculated for both positive and negative emotions and represented by the standard deviation of the mean level of state emotion (Grühn et al., 2013). In terms of direction, a larger standard deviation represented more EV.

*Appraisals* included motivational relevance: “How important is the valued action for you to take?” rated on a 10‐point Likert scale (adapted from Verduyn et al., 2013); perceived difficulty of initiating action: “How difficult is it for you to initiate this action?” rated on a 10‐point Likert scale; and perceived action self‐efficacy: “To what extent do you believe that you will be able to initiate this action?” rated on a 7‐point Likert scale.

Participants’ *action tendencies* were measured with a recently validated nonverbal pictorial measure: Depicted Action Tendencies (DAT; OToole & Mikkelsen, 2021). The DAT items portray characters engaged in actions reflecting four different action tendencies: approach a reward (e.g., running toward something meaningful/rewarding), approach a threat (e.g., confronting someone), avoid a reward (e.g., withdrawal from meaningful activities), or avoid a threat (e.g., fleeing from something or freezing; see Table 1). There were two items for each of the four action tendency configurations: *Approach Reward* (DAT approach reward 1/DAT approach reward 2), *Approach Threat* (DAT approach threat 1/DAT approach threat 2), *Avoid Threat* (DAT avoid threat 1/DAT avoid threat 2), and *Avoid Reward* (DAT avoid reward 1/DAT avoid reward 2). Participants were asked to rate the extent to which they felt like the character(s) in these pictures on a 5‐point Likert scale. All subscales except for the scale comprised of the two “approach threat” items had Cronbach's alpha values that exceeded .7, and items were correlated of large magnitude (*r*s= .5–.8). Accordingly, we analyzed the two items of “approach threat” separately, while the other three motivational configurations were summed (see Table 1).

**TABLE 1:** Depicted Action Tendencies (DAT) material

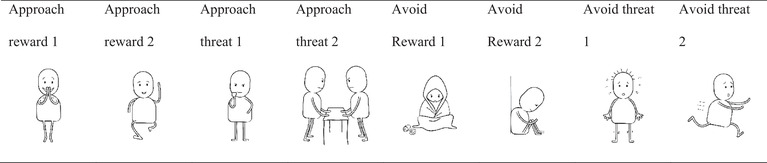

*Completed valued action* (yes/no) was a secondary outcome and assessed orally during a follow‐up phone call, where participants were asked if the action had actually been completed.

### 2.5 Analytic strategy

Baseline differences were investigated with one‐way ANOVAs. All hypotheses were explored using a repeated‐measures ANOVA, conducting a 2 (time: pre, post) × 3 (group conditions: EXP, CON, N) interaction analysis. Effect sizes were reported using partial eta squared (Richardson, 2011), with values of .01, .06, and .14 denoting small, medium, and large effect sizes, respectively. Significant interaction analyses were followed up by post hoc comparisons, employing a *p*‐correction corresponding to the three comparisons for each outcome variable (.05/3 = .017).

## 3 RESULTS

Baseline characteristics for participants can be found in Table 2. There were no statistically significant differences between groups regarding age, gender, or trait positive/negative emotions. All participants followed the instructions appropriately, held the postures, and engaged in the movements as requested.

**TABLE 2:** Baseline characteristics: Means (*M*) and standard deviations (*SD*) in all groups

	EXP (N = 30)	CON (N = 29)	N (N = 31)	Group difference
Age	23.77 (2.64)	24.62 (3.12)	24.90 (4.07)	F = 0.9, p = .395
Women	21	24	25	χ^2^ = 1.6, p = .446
Trait PA	35.43 (6.32)	36.14 (7.25)	35.29 (6.50)	F = 0.1, p = .873
Trait NA	18.07 (6.07)	19.41 (5.91)	19.35 (5.87)	F = 0.5, p = .614

Abbreviations: NA, Negative Affect (PANAS); PA, Positive Affect (PANAS).

### 3.1 Positive and negative emotions

We investigated if the groups differed in state positive emotions and negative emotions and found an interaction effect for positive emotions (*F*(1, 87) = 3.74, *p* = .028, *η*_p_^2^ = .08). Exploring this finding further, the difference between the EXP and neutral control condition was statistically significant, favoring the EXP condition of a large magnitude (*F*(1, 59) = 9.24, *p* = .004, *η*_p_^2^ = .14). The CON and neutral control conditions also differed from each other, favoring the CON condition of a medium magnitude (*F*(1, 58) = 4.12, *p* = .047, *η*_p_^2^ = .07), albeit not statistically significant at the corrected *p*‐level. The two active conditions did not differ (*F*(1, 57) = 0.14, *p* = .713, *η*_p_^2^ < .01). Regarding state negative emotions, the 2 × 3 interaction analysis was nonsignificant.

### 3.2 Emotion experience

**FIGURE 1:**
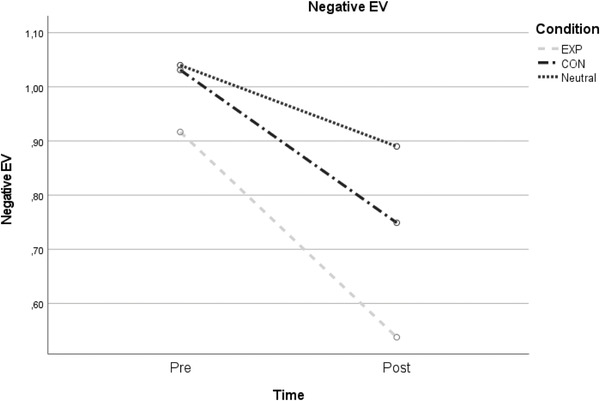
Time × group interaction for negative emotion variability

The overall 2 × 3 interaction analysis was significant (*F*(1, 87) = 3.35, *p* = .040, *η*_p_^2^ = .07). A significant effect of a medium magnitude was found for group differences between the EXP and neutral control (*F*(1, 59) = 6.20, *p* = .016, *η*_p_^2^ = .10), showing that EV decreased the most in the EXP condition. No differences were detected when comparing the CON and neutral control conditions (*F*(1, 58) = 1.96, *p* = .167, *η*_p_^2^ = .03) or the EXP and CON conditions (*F*(1, 57) = 1.41, *p* = .240, *η*_p_^2^ = .02) (see Table 3 and Figure 1). Concerning positive EV, no significant interaction effects were detected.

**TABLE 3:** Means (*M*), standard deviations (*SD*), and main and interaction effects pre‐ and postmanipulation

Dependent variables	EXP (N = 30) M (SD)		CON (N = 29) M (SD)			N (N = 31) M (SD)		Main effect (pre, post)			Group (EXP, CON, N) × time (pre, post)	
	Pre	Post	Pre	Post	Pre	Post	F	p	η_p_^2^	F	p	η_p_^2^
Total state PA	27.77 (7.36)	31.73 (7.49)	22.14 (7.96)	25.48 (9.16)	27.90 (11.07)	28.06 (11.08)	16.38	<.001*	.16	3.74	.028*	.08
EXP vs. CON										0.14	.713	<.01
EXP vs. N										9.24	.004*	.14
CON vs. N										4.12	.047	.07
Total state NA	18.87 (5.79)	14.40 (3.84)	19.83 (6.88)	16.14 (5.42)	20.71 (7.48)	19.13 (6.98)	39.72	<.001*	.31	2.86	.063	
EXP vs. CON										0.36	.551	
EXP vs. N										6.88	.011*	
CON vs. N										2.35	.131	
Positive EV	0.89 (0.28)	0.84 (0.32)	0.88 (0.42)	0.75 (0.38)	0.73 (0.30)	0.70 (0.33)	5.75	.019*	.06	1.08	.344	.24
EXP vs. CON										1.06	.308	.02
EXP vs. N										0.09	.770	<.01
CON vs. N										1.78	.187	.03
Negative EV	0.92 (0.41)	0.54 (0.33)	1.03 (0.42)	0.75 (0.45)	1.04 (0.56)	0.89 (0.55)	54.56	<.001*	.39	3.35	.040*	.07
EXP vs. CON										1.41	.240	.02
EXP vs. N										6.20	.016*	.10
CON vs. N										1.96	.167	.03
Appraisal: Self‐efficacy	4.67 (1.42)	5.63 (0.89)	4.28 (1.67)	5.14 (1.71)	5.00 (1.60)	5.35 (1.54)	27.72	<.001*	.24	1.90	.155	.04
EXP vs. CON										0.11	.745	<.01
EXP vs. N										3.12	.083	.05
CON vs. N										2.14	.149	.04
Appraisal: Difficulty task	7.73 (1.41)	5.97 (2.31)	7.83 (6.55)	6.55 (2.05)	7.55 (1.80)	6.97 (2.40)	29.36	<.001*	.25	2.43	.094	.05
EXP vs. CON										0.87	.355	.02
EXP vs. N										4.54	.037	.07
CON vs. N										1.58	.213	.03
Appraisal: Importance task	8.10 (1.30)	8.07 (1.78)	8.31 (1.26)	8.34 (1.32)	8.65 (0.95)	8.45 (1.46)	0.24	.629	.00	0.26	.769	.01
EXP vs. CON										0.04	.846	<.01
EXP vs. N										0.21	.651	<.01
CON vs. N										0.76	.388	.01
DAT approach threat 1	3.00 (1.29)	3.80 (1.03)	2.14 (1.13)	2.44 (1.24)	2.68 (1.35)	2.74 (1.55)	12.95	.001*	.13	3.27	.043*	.07
EXP vs. CON										1.80	.186	.03
EXP vs. N										6.52	.013*	.10
CON vs. N										1.42	.239	.02
DAT approach threat 2	2.40 (1.52)	2.40 (1.13)	2.48 (1.15)	2.24 (1.18)	2.10 (1.38)	1.87 (1.12)	1.60	.210	.02	0.40	.671	.01
EXP vs. CON										0.54	.467	.01
EXP vs. N										0.61	.439	.01
CON vs. N										<0.01	.957	<.01
DAT approach reward 1 + 2	4.47 (2.06)	5.43 (2.27)	3.24 (1.66)	4.07 (2.36)	4.26 (2.25)	4.58 (2.44)	15.84	<.001*	.15	1.24	.294	.03
EXP vs. CON										0.10	.755	<.0
EXP vs. N										2.23	.141	.04
CON vs. N										1.40	.242	.02
DAT avoid reward 1 + 2	3.50 (1.93)	2.77 (1.50)	4.13 (2.26)	3.34 (2.11)	4.52 (2.42)	4.13 (2.54)	17.69	<.001*	.17	0.71	.497	.02
EXP vs. CON										0.04	.849	<.01
EXP vs. N										0.78	.382	.01
CON vs. N										1.04	.313	.02
DAT avoid threat 1 + 2	5.27 (2.26)	3.07 (1.41)	5.90 (2.26)	4.24 (1.72)	5.39 (2.73)	4.94 (2.64)	48.42	<.001*	.36	6.39	.003*	.13
EXP vs. CON										1.41	.241	.02
EXP vs. N										11.16	.001*	.16
CON vs. N										5.20	.026	.08

*Note*: Significant results from the main and 2 × 3 interaction analyses were considered statistically significant at p < .05. In the post hoc group comparisons (marked in italics), a p‐level <.017 was considered statistically significant. Significant results are marked with an asterisk (*).

Abbreviations: CON, contractive group; EV, emotion variability; EXP, expansive group; N, neutral control group (usual) display; NA, negative emotions; PA, positive emotions. Figure 2, 3

**FIGURE 2:**
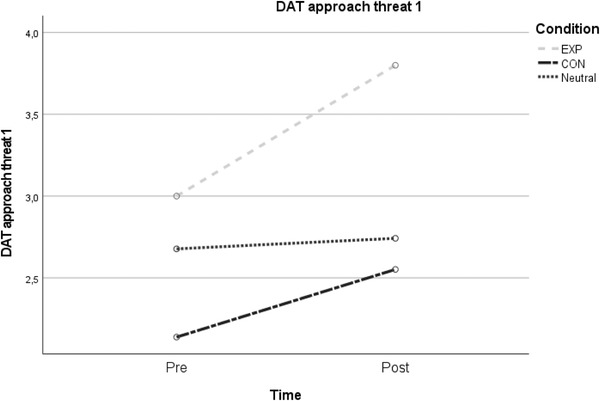
Time × group interaction for approach threat motivation

**FIGURE 3:**
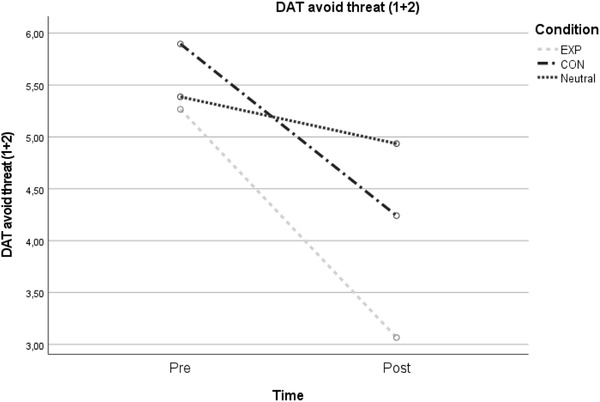
Time × group interaction for avoid threat motivation

### 3.3 Appraisals

Regarding appraisals, the 2 × 3 interaction analyses were all nonsignificant.

### 3.4 Action tendencies

Concerning approach tendencies, the 2 × 3 interaction analysis on DAT “approach threat 1” was significant (*F*(1, 87) = 3.27, *p* = .043, *η*_p_^2^ = .07). Exploring this finding further, post hoc analyses revealed a significant interaction effect on DAT “approach threat 1” only when comparing the EXP and neutral control condition (*F*(1, 59) = 6.52, *p* = .013, *η*_p_^2^ = .10). No significant effects were found for DAT “approach threat 2.”

Regarding DAT avoid threat (1 + 2), the overall 2 × 3 interaction analysis was significant (*F*(1, 87) = 6.39, *p* = .003, *η*_p_^2^ = .13). Comparing groups post hoc, there were significant interaction effects when comparing the EXP and neutral control conditions (*F*(1, 59) = 11.16, *p* = .001, *η*_p_^2^ = .16). The difference between the CON and neutral control condition (*F*(1, 58) = 5.20, *p* = .026, *η*_p_^2^ = .08) did not meet the corrected *p*‐level. Regarding DAT approach reward (1 + 2) and DAT avoid reward (1 + 2), no significant interaction effects were detected (see Table 3).

### 3.5 Completed valued action

Concerning valued actions completed, 50% of the participants took action in the EXP condition, 37% in the CON condition, and 32% in the neutral control condition. When comparing groups, there were no statistically significant group differences (*χ* ^2^(2) = 2.5, *p* = .281).

## 4 DISCUSSION

We wanted to investigate the effects of two different bodily manipulations (i.e., EXP and CON), compared to a control group, on specific emotional processes believed to be involved in preparing, motivating, and taking a valued action. First, for four out of the 12 outcomes, a significant interaction effect was detected. When contrasting conditions for these four effects, they seemed to be driven by the EXP condition outperforming the neutral control condition but not the CON condition. Second, several main effects were found. In the absence of the expected interaction effect, these results suggest that simply identifying a valued action and explicating the underlying motivational conflict may be beneficial.

These findings run somewhat counter both to our hypotheses and to the findings in recent meta‐analyses, showing that effects of bodily displays may be ascribed to the absence of contractive displays rather than the presence of expansive displays (Elkjær et al., 2022; Körner et al., 2022). However, previous experiments have not evaluated the effect of bodily displays within the context of personal relevance. Should our findings be replicated, it could point to the potential of expansive displays as opposed to neutral displays being advantageous when in a motivational conflict in which one wants to take a valued action.

The most robust findings across outcomes, however, are the main effects. Based on these, one could argue that *any* type of bodily displays may hold a potential to enhance approach behavior in the context of a motivational conflict. The *congruency hypothesis* could assist in this explanation. It states that bodily displays should be congruent with the experienced affective and motivational states in order to facilitate beneficial affective and behavioral outcomes (Riskind, 1984; Riskind & Gotay, 1982; Welker et al., 2013). Indeed, past studies have shown that *incongruent* conditions (e.g., expansive displays during failure experiences and contractive displays during success experiences) caused significant more negative outcomes than the *congruent* conditions (e.g., contractive displays during failure experiences and expansive displays during success experiences; for a review, see Elkjær et al., 2022). As such, congruency between bodily displays and emotional states may be important when using the body to change affect and behavior. This could imply adopting a contractive display when experiencing avoidance or withdrawal tendencies and adopting an expansive display when experiencing approach tendencies. When sad or scared of taking action, it has been proposed that connecting with those emotions and associated motivations may enhance emotional processing, allowing the individual to more fully acknowledge what is at stake in the situation, which may in itself diminish avoidance tendencies (Greenberg, 2004; Timulak & Pascual‐Leone, 2015). As such, contractive displays may be believed to assist in such processing, a hypothesis that needs further investigation. An alternative explanation could simply be that there may have been an overall demand effect across conditions. Specifically, participants attended an experimental study, talked about personal conflicts, and may have tried to act in a socially desirable way, that is, move toward their previously stated goals.

A number of methodological characteristics should be taken into account. First, participants were being observed by the experimenter. Previous findings have indicated that individuals may rely less on internal cues in making judgments when feeling monitored or observed (Noah et al., 2018). This could potentially have masked the effects of bodily displays and a replication study should take this into account. Second, participants were not instructed to repeat the regulation strategies before planning to engage in the difficult task in real life. This could potentially have enhanced the chance of taking action. Third, actual action taken was measured categorically (yes/no), thus potentially not capturing instances in which the action was partly completed. In addition, future research is advised not only to assess the degree of success of taking action (e.g., on a Likert scale), but also action tendencies, emotion experience, and appraisals as there may be several reasons why actual action is not taken despite the individual wanting to take the action.

Turning to the practical and clinical utility, the finding that different bodily displays may facilitate approach toward something valued is promising for clients with difficulties employing more traditional regulatory (e.g., attentional) strategies. Individuals struggling with taking valued action either because of active avoidance (prototypical of anxiety disorders; APA, 2013) or passivity and withdrawal (prototypical of depressive disorders; APA, 2013) may benefit from these findings. Specifically, alterations of bodily displays could potentially be used in already existing therapies such as *behavioral activation* for depressed individuals, where the main purpose for the client is, despite lack of energy and motivation, to identify valuable activities and initiate behavior accordingly (Lejuez et al., 2011). Based on our results that expansive bodily displays may help prepare to take action in certain instances, compared with adopting a neutral display, the integration of bodily manipulations into existing therapies aiming for action initiation might be worthwhile investigating.

### 4.1 Limitations

First, a *p*‐correction was employed for the post hoc analyses (*p* = .017) corresponding to the number of comparisons within each outcome. Second, as a control condition, we asked participants to mentally process the valued action while assuming neutral bodily displays. One could claim that thinking of a task in a certain way is a rather active strategy in and of itself. However, this was our attempt to isolate the effects of the bodily displays while maintaining a focus on an ideographic task. In relation to this, more aspects than expansive/contractive displays were varied than in the control condition. For example, the control group sat throughout the study, whereas the experimental groups sat and stood. However, making the individual stand would quickly come to resemble an active control group. Future research may need to include both passive and active control groups in order to more comprehensively evaluate the specificity of the displays in question. Third, in the expansive condition, the participants nodded their heads, whereas they shook their heads in the contractive condition and more than the posture was thus manipulated. While this decision detracts from the ability to conclude which elements of the manipulation were more or less effective, they were combined in an attempt to maximize the potential effect. Furthermore, it is possible that these nonverbal movements may have further triggered participants’ expectations and hypotheses about the purpose of the experiment and their action tendencies. Fifth, to consolidate that the context of a personal relevant task plays a role, it may be contrasted against a separate condition in which an unimportant task could be utilized. Sixth, we were unable to test the robustness of the results by running the analyses with and without participants with knowledge of power posing. Seventh, we only included healthy participants and the findings need to be further explored within clinical populations.

## 5 CONCLUSIONS

In conclusion, only a few hypothesized significant interaction effects were observed. In probing these effects, results suggested that expansive bodily displays outperformed the control group, leading to more positive emotions, more approach action tendencies, less negative EV, and less avoidance action tendencies toward threat. Contrary to our expectation, contractive displays did not reveal less approach behavior than expansive or neutral displays, rather several main effects were detected. This conclusion runs somewhat counter both to our hypotheses and to the findings in recent meta‐analytic work. However, previous experiments have not evaluated the effect of bodily displays within a personally relevant context.

## AUTHOR CONTRIBUTIONS

Mia S. O'Toole and Emma Elkjær conceptualized and designed the study. Emma Elkjær curated the data. All authors analyzed and interpreted the results. All authors wrote the original draft. All authors reviewed the results and approved the final version of the manuscript.

## CONFLICT OF INTEREST

The authors declare no conflict of interest.

### PEER REVIEW

The peer review history for this article is available at https://publons.com/publon/10.1002/brb3.2855

## Supporting information

Appendix Full Scripts

## Data Availability

The data that support the findings are available upon request.

## Note

i A number of additional questionnaires were obtained at baseline but were not relevant for the current study aims: These included worry (Brief PSWQ; Topper et al., 2014), rumination (RRS; Treynor et al., 2003), reappraisal and expressive suppression (ERQ; Gross & John, 2003), acceptance and action (AAQ; Bond & Bunce, 2003).
