# Supplementary material for: Using bodily displays to facilitating approach action outcomes within the context of a personally relevant task
Source: Brain Behav. 2022 Dec 26;13(1):e2855. doi: 10.1002/brb3.2855 (PMC9847621; doi:10.1002/brb3.2855)
Supplement: Supplementary file 1 — Appendix Full Scripts [file BRB3-13-e2855-s001.docx]

**Appendix**

**Full Scripts**

***The EXP bodily regulation strategy***

*When you are ready, then close your eyes and imagine – as vivid as possibly – the situation which we have identified together. Imagine exactly the moment, where it is most difficult for you to take action. Right there where the anxiety, the nervousness or worry occur. Notice your surroundings (…) Where are you? Who are there with you? What do you see and feel? Throughout this exercise, try to focus on this specific moment, as vivid as you can (…). In the next minutes, you will be reminded of focusing on this very moment with the sentence: “Remember the moment”.*

*(Pause 20 sec).*

*Stay seated in an upright position. Almost as if someone gently pulls your head upwards. While you stay in this position, notice your face. Relax in the muscles around your eyes (…), and forehead (…).Try to smile gently. Stay seated and nod your head. Up and down. Up and down (…).*

*Remember the moment (Pause 10 Sec).*

*Now, put both of your palms on the back of your head with your elbows to the side, in a way where you push your chest a bit forward. Lift your chin as well.*

*Remember the moment (Pause 10 Sec).*

*Put down your hands. Open your eyes, and when you are ready: Stand up (…) and adopt an upright position. Make sure that your shoulders are pulled backwards (…). Bent your arms and place both of your hands on your hips. Do not collapse. Lift your chin gently and push your chest forward. Stand this way for a little while (…)*

*Remember the moment (Pause 10 Sec).*

*When you are ready, take a seat once again, and then this exercise has finished.*

***The CON bodily regulation strategy***

*When you are ready, then close your eyes and imagine – as vivid as possibly – the situation which we have identified together. Imagine exactly the moment, where it is most difficult for you to take action. Right there where the anxiety, the nervousness or worry occur. Notice your surroundings (…) Where are you? Who are there with you? What do you see and feel? Throughout this exercise, try to focus on this specific moment, as vivid as you can (…). In the next minutes, you will be reminded of focusing on this very moment with the sentence: “Remember the moment”.*

*(Pause 20 sec).*

*Stay seated in a slumped position. Almost as if someone gently pulls your shoulders forwards. While you stay in this position, notice your face. Relax in the muscles around your eyes (…), and forehead (…). Stay seated and shake your head. From side to side. Side to side (…).*

*Remember the moment (Pause 10 Sec).*

*Open your eyes, and when you are ready: Stand up (…) and adopt a slumped position. Make sure that your shoulders are pulled forwards (…). Let your arms hang limply down. It is okay to let your body collapse a little. Lower your chin gently (...) Stand this way for a little while (…)*

*Remember the moment (Pause 10 Sec).*

*Now, put right hand on your left shoulder and your left hand on your right shoulder (…) and double up by leaning your back a little forward. Lower your chin so that you bow your head. Remember the moment (Pause 10 Sec).*

*When you are ready, take a seat once again, and then this exercise has finished.*

***The control strategy***

*When you are ready, then close your eyes and imagine – as vivid as possibly – the situation which we have identified together. Imagine exactly the moment, where it is most difficult for you to take action. Right there where the anxiety, the nervousness or worry occur. Notice your surroundings (…) Where are you? Who are there with you? What do you see and feel? Throughout this exercise, try to focus on this specific moment, as vivid as you can (…). In the next minutes, you will be reminded of focusing on this very moment with the sentence: “Remember the moment”.*

*Sit like this (…) Sit as you find it naturally. Remember the moment.*

*(Pause 30 Sec).*

*Observe what is going on in your mind, while sitting here and remembering the moment.*

*(Pause 30 Sec).*

*When you are ready, open your eyes, and then this exercise has finished.*
